# Circulating α-Klotho Levels in Relation to Cardiovascular Diseases: A Mendelian Randomization Study

**DOI:** 10.3389/fendo.2022.842846

**Published:** 2022-02-07

**Authors:** Xingang Sun, Lu Chen, Yuxian He, Liangrong Zheng

**Affiliations:** Department of Cardiology and Atrial Fibrillation Center of The First Affiliated Hospital of Zhejiang University, Hangzhou, China

**Keywords:** α-klotho, coronary artery disease, myocardial infarction, atrial fibrillation, heart failure, stroke, Mendelian randomization

## Abstract

**Background:**

Several studies have reported a protective role of circulating α-Klotho on cardiovascular diseases (CVD); however, the causality remains unclear. We aim to elucidate whether genetically predicted circulating α-Klotho levels were causally associated with the risk of coronary artery disease (CAD), atrial fibrillation (AF), heart failure (HF), stroke, ischemic stroke (IS), and IS subtypes.

**Methods:**

A two-sample Mendelian randomization (MR) study was designed, with 5 single-nucleotide polymorphisms associated with circulating α-Klotho levels utilized as instrumental variables. MR estimates on each CVD outcome derived from the fixed-effects inverse-variance weighted (IVW) approach in different data sources were combined by the fixed-effects meta-analysis approach, complemented by several sensitivity analyses including the simple median, the weighed median, MR-Egger regression, and MR-pleiotropy residual sum and outlier.

**Results:**

In the meta-analysis combining different data sources, suggestive inverse causal association of circulating α-Klotho concentrations with CAD [Odds ratio (OR), 0.97; 95% confidence interval (CI), 0.94, 1.00; P = 0.044] and significant inverse association of circulating α-Klotho concentrations with AF (OR, 0.96; 95% CI, 0.93, 0.99; P = 0.005) was observed. However, there was no causal association of α-Klotho with HF, any stroke, IS, or IS subtypes neither in different data sources nor in the meta-analysis. Complementary sensitivity analyses showed consistent and robust results in general.

**Conclusion:**

Evidence was found for a protective effect of circulating α-Klotho on the prevention of AF risk. However, no significant causal association between genetically predicted circulating α-Klotho levels and risk of CAD, HF, stroke, IS, or IS subtypes was found.

## Introduction

α-Klotho (also simply referred to as Klotho) was originally identified as an aging suppressor gene. In 1997, Kuro-o et al. fortuitously discovered that Klotho-deficient mice developed several symptoms resembling human aging, including shortened lifespan, arteriosclerosis, and multiple organ degeneration (1). A few years later, Kurosu et al. further demonstrated that overexpression of Klotho in transgenic mice extended the life span and Klotho protein could act as a circulating hormone (2).

The α-Klotho gene encodes a single-pass transmembrane protein, which is mainly expressed in the kidney distal tubules, the brain choroid plexus, and the parathyroid gland. There are two forms of α-Klotho: membrane and soluble form, and each form has different functions (3). The membrane-bound α-Klotho protein functions as an obligatory co-receptor for endocrine fibroblast growth factor 23 (FGF23), thus regulating vitamin D metabolism and phosphate homeostasis (4). The soluble α-Klotho, also referred to as circulating α-Klotho, is released by cleavage of the extracellular domain of the transmembrane α-Klotho and is the main functional form in the circulation (5).

In recent years, scientists have focused on the role of circulating α-Klotho in the risk of cardiovascular diseases (CVD). Several studies reported a protective role of α-Klotho on CVD, with circulating α-Klotho levels inversely associated with the risk of CVD, coronary artery disease (CAD), and atrial fibrillation (AF) (6–8). Although these studies provided a novel sight into preventing the development of CVD, they were potentially biased by reverse causation and confounding factors. In addition, no study has investigated the causal relationships between circulating α-Klotho concentrations and the risk of CVD yet.

The Mendelian randomization (MR) approach utilizes genetic variants as instrumental variables (IVs) for a trait (exposure) to explore the causal associations between exposure and explicit diseases (outcomes). As genetic variants were unlikely to be affected by disease status, the MR method avoids reverse causation in observational studies (9). Moreover, under the assumption that exposure-associated genetic variants were randomly assigned during conception, genetic variants which have a specific effect on circulating α-Klotho levels could be used as IVs to assess associations with CVD outcomes, independent of confounding factors (9). In a recent genome-wide association study (GWAS) meta-analysis, Gergei et al. identified six single-nucleotide polymorphisms (SNPs) genome-wide significantly associated with circulating α-Klotho levels, enabling us to apply the MR method to investigate the causal association of circulating α-Klotho levels with CVD risk. Therefore, we conducted a two-sample MR study to elucidate whether genetically predicted circulating α-Klotho levels were causally associated with the risk of 8 CVD outcomes, including CAD, AF, heart failure (HF), stroke, ischemic stroke (IS), and IS subtypes.

## Methods

### Study Design

**Figure 1:**
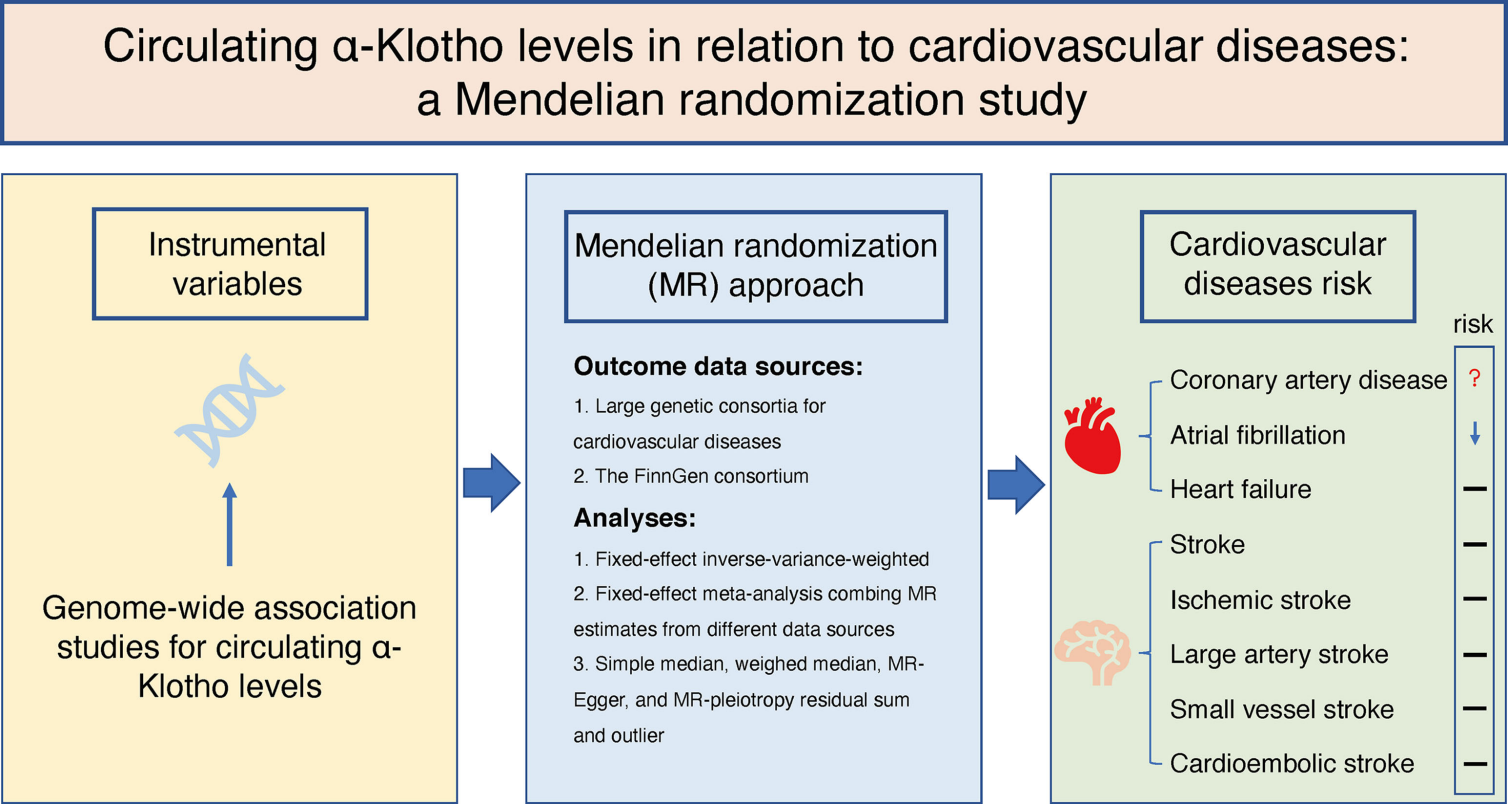
Schematic representation of this two-sample Mendelian randomization study.

We designed the present two-sample MR study to evaluate the causal inference between circulating α-Klotho concentrations and CVD based on summary-level data obtained from publicly available GWASs (**Figure 1**). This MR study was based on three principal assumptions. First, the genetic variants selected as IVs were strongly associated with circulating α-Klotho levels. Second, the IVs were not associated with any risk factors. Third, the genetic variants directly affected the CVD outcomes only through circulating α-Klotho levels rather than other pathways.

### Outcome Data Sources

8 CVD endpoints were set as outcomes in our study with the case number ranging from 4,373 [large artery stroke (LAS)] to 60,801 (CAD). Summary-level data for CVD outcomes were obtained from Coronary ARtery DIsease Genome-wide Replication and Meta-analysis (CARDIoGRAM) plus The Coronary Artery Disease (C4D) Genetics (CARDIoGRAMplusC4D) consortium (10), GWAS meta-analysis by Nielsen et al. (11), Heart Failure Molecular Epidemiology for Therapeutic Targets (HERMES) consortium (12), MEGASTROKE consortium (13), and the FinnGen study (14), respectively. GWAS studies included in the present MR study have obtained ethical approval from respective institutional review boards and all participants involved have provided informed consents. Detailed data on involved outcome data sources were presented in **Supplementary Table 1**.

### Instrumental SNPs Selection

A meta-analysis of GWAS including 4,376 European individuals identified six independent SNPs associated with circulating α-Klotho concentrations with genome-wide significance (P < 5 × 10^-8^) (15). Among the six SNPs, one SNP (rs532436) was excluded from this MR study due to pleiotropic effects found in Phenoscanner database (16) on low density lipoprotein (P = 4.02 × 10^-30^), total cholesterol (P = 6.14 × 10^-26^), CAD (P = 4.39 × 10^-14^), and diastolic blood pressure (P = 5.98 × 10^-11^) (**Supplementary Table 2**), leaving the remaining 5 SNPs utilized as instrumental variables (IVs). F-statistic was calculated to assess the strength of each SNP with the formula of F = R^2^ (N − 2)/(1 − R^2^), where R^2^ refers to the proportion of variance explained by IVs and N stands for the sample size (17). If the selected SNPs were not available in the summary statistics of outcome data sources, proxy SNPs (r^2^ > 0.8) would be searched to replace them in an online website (http://snipa.helmholtz-muenchen.de/snipa3/).

### Statistical Analysis

We employed the fixed-effects meta-analysis approach to combine MR estimates on each CVD outcome derived from the fixed-effects inverse-variance-weighted (IVW) approach in different data sources. Sensitivity analyses, including the simple median, the weighted median (18), MR-Egger regression (19), and MR-pleiotropy residual sum and outlier (MR-PRESSO) (20) were performed as complementary analyses to evaluate the robustness of the results and detect possible pleiotropy. Heterogeneity among IVs was calculated by Cochrane’s Q in IVW models, and if there was evidence of heterogeneity, the random-effects IVW models would be applied. P-values for the intercept in MR-Egger regression were used to indicate horizontal pleiotropy. As MR-Egger regression can generate estimates after correction for pleiotropy, this method would be preferred to evaluate the causal associations in the case of P_intercept_ < 0.05 (19). We also applied the global test and outlier test in MR-PRESSO to detect potential pleiotropy and outlier. Moreover, to avoid missing the information of SNP rs532436, we performed heterogeneity and pleiotropy test indicated by Cochrane’s Q and MR-PRESSO analyses with all six α-Klotho-associated SNPs in the large genetic consortia for CVD outcomes (rs532436 was not available in the FinnGen consortium and no proxy SNP was found). All presented odds ratios (ORs) and the corresponding confidence intervals (CIs) were scaled to per standard deviation (SD) increase in genetically predicted circulating α-Klotho concentrations. Given that there were 8 CVD outcomes involved in the present study, associations with P-values < 0.006 were considered as significant associations. Associations with P-values between 0.006 and 0.05 were regarded as suggestive associations. All analyses were performed using the TwoSampleMR (21) and MRPRESSO (20) packages in R Software 4.0.2.

## Results

The 5 SNPs explained 7.68% of the variance in circulating α-Klotho concentrations (**Table 1**). The F-statistic for each SNP was ≥ 30, indicating that it was strong enough to avoid weak instrument bias (**Table 1**). Rs8176672 could not be directly obtained from the FinnGen data of SNP-CVD associations and its proxy SNP (rs1137827, r^2^ = 1) was identified. Characteristics of the genetic associations of instruments with CVD outcomes were presented in **Supplementary Table 3**.

**Table 1:** Information on instrumental SNPs associated with circulating α-Klotho levels.

SNPs	Chr	Position (hg19)	EA/NEA	EAF	R^2^ (%)	F	Beta	SE	P-value
rs12607664	18	24693221	T/G	0.316	2.54	114.1	0.243	0.022	2.28×10^-27^
rs8176672	9	136142185	T/C	0.072	2.20	98.3	0.406	0.041	2.11×10^-23^
rs532436*	9	136149830	G/A	0.240	1.41	62.3	0.204	0.026	5.86×10^-15^
rs1056008	12	662838	C/T	0.268	1.32	58.6	0.184	0.024	1.80×10^-14^
rs7333961	13	33533269	A/G	0.046	0.94	41.6	-0.327	0.051	1.73×10^-10^
rs881301	8	38332318	C/T	0.413	0.68	30.0	-0.119	0.021	2.23×10^-08^

*SNP rs532436 was excluded from the primary analyses due to pleiotropic effects.

SNPs, single-nucleotide polymorphisms; Chr, chromosome; EA, effect allele; NEA, non-effect allele; EAF, effect allele frequency; SE, standard error; R^2^, the variance explained by the SNP; F, indicating the F-statistic, was calculated as follows: F = (N − 2) × R^2^/(1 − R^2^), where N stands for the sample size of 4,376.

**Figure 2:**
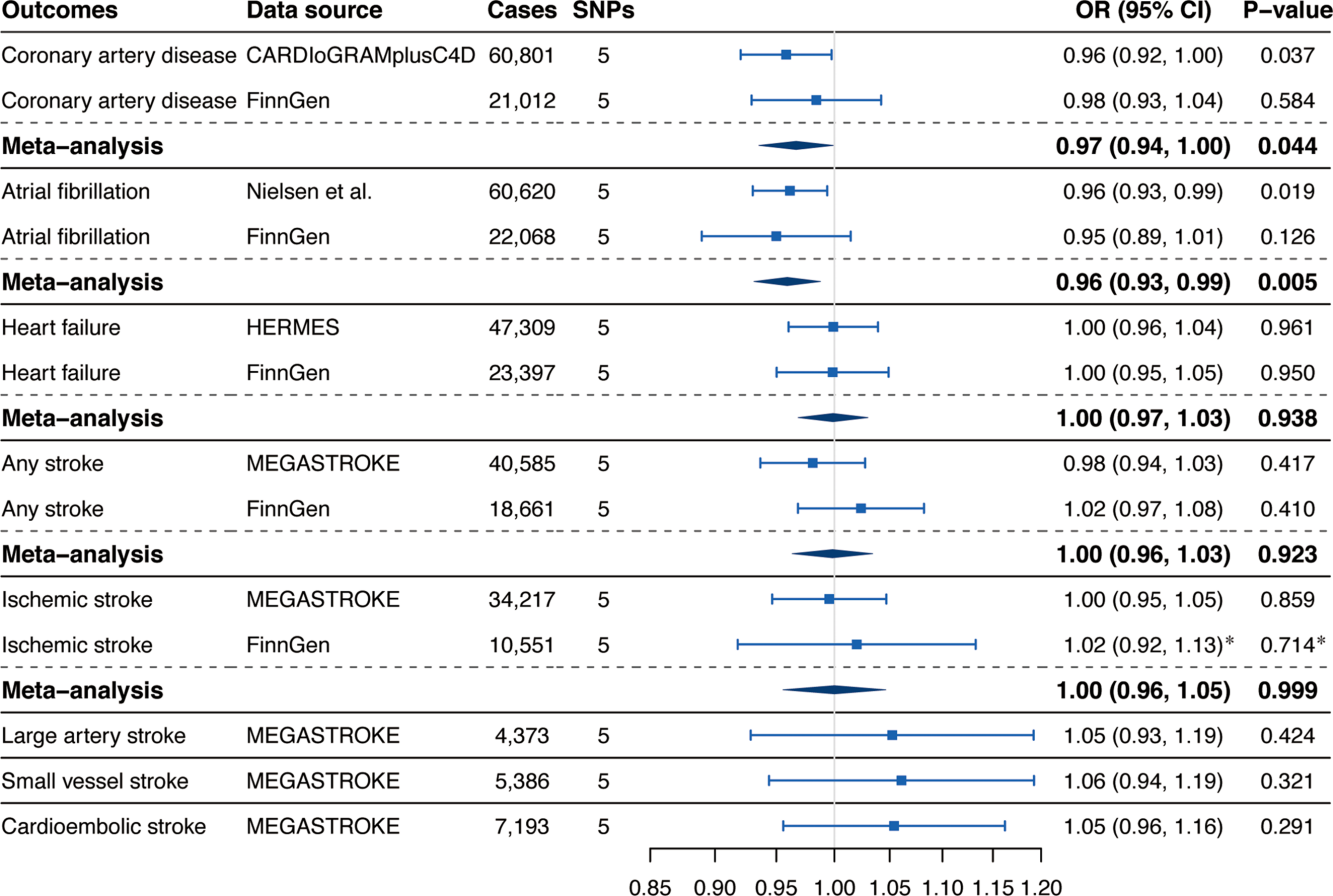
Associations of genetically predicted circulating α-Klotho levels with cardiovascular diseases. SNPs, single-nucleotide polymorphisms; OR, odds ratio; CI, confidence interval; CARDIoGRAMplusC4D, Coronary ARtery DIsease Genome-wide Replication and Meta-analysis (CARDIoGRAM) plus The Coronary Artery Disease (C4D) Genetics; HERMES, Heart Failure Molecular Epidemiology for Therapeutic Targets. *Results were obtained from the multiplicative random-effects inverse-variance weighted method.

Associations of genetically predicted circulating α-Klotho concentrations with CVD risk were displayed in **Figure 2**. We observed suggestive inverse associations of circulating α-Klotho levels with the risk of CAD in the CARDIoGRAMplusC4D (OR, 0.96; 95% CI, 0.92, 1.00; P = 0.037; **Figure 2**) and AF in the GWAS meta-analysis by Nielsen et al. (OR, 0.96; 95% CI, 0.93, 0.99; P = 0.019; **Figure 2**). Although the associations for CAD and AF were not replicated in the FinnGen consortium, the suggestive and significant associations for CAD and AF persisted in the meta-analysis combining different data sources (CAD: OR, 0.97; 95% CI, 0.94, 1.00; P = 0.044; AF: OR, 0.96; 95% CI, 0.93, 0.99; P = 0.005; **Figure 2**). Genetically predicted α-Klotho levels were not associated with HF, any stroke, IS, LAS, small vessel stroke (SVS), or cardioembolic stroke (CES) neither in different data sources nor in the meta-analysis (**Figure 2**).

**Figure 3:**
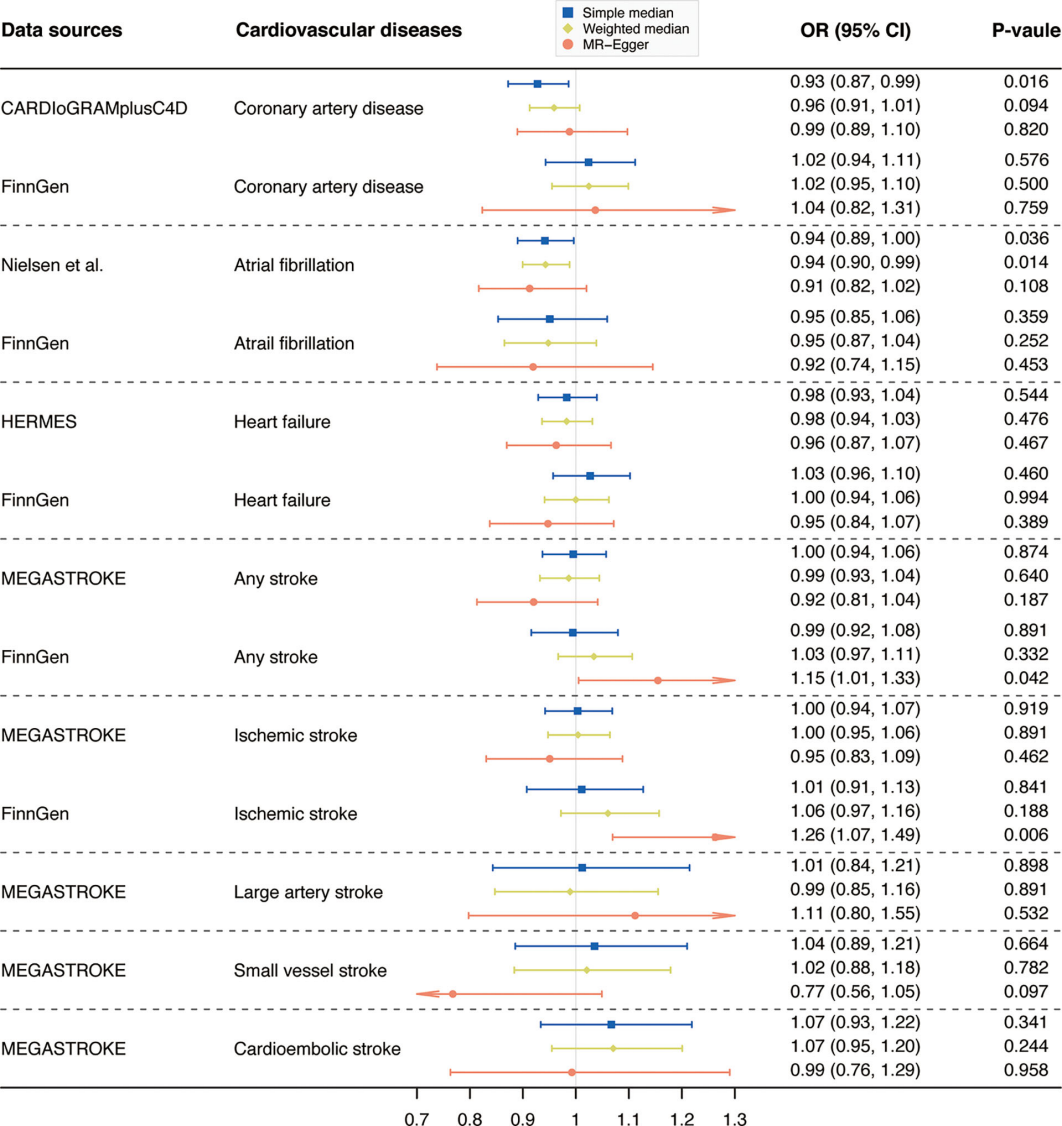
Complementary analyses of the association between circulating α-Klotho levels and cardiovascular diseases. OR, odds ratio; CI, confidence interval; CARDIoGRAMplusC4D, Coronary ARtery DIsease Genome-wide Replication and Meta-analysis (CARDIoGRAM) plus The Coronary Artery Disease (C4D) Genetics; HERMES, Heart Failure Molecular Epidemiology for Therapeutic Targets. Note: as no outlier was identified by Mendelian randomization-pleiotropy residual sum and outlier (MR-PRESSO), results from MR-PRESSO were not shown in this figure.

Results from complementary analyses showed that the suggestive protective effect of α-Klotho on CAD in the CARDIoGRAMplusC4D remained in the simple median analysis but was weakened in the weighted median and MR-Egger methods (**Figure 3**). Compared with the estimate obtained in IVW in the AF GWAS meta-analysis by Nielsen et al. the results were consistent and robust in the simple median and weighted median methods, but not in the MR-Egger method which has broader CIs (**Figure 3**). Whereas, the MR estimates for AF in the FinnGen study were stable across all complementary analyses (**Figure 3**). Genetically predicted α-Klotho concentrations were also not significantly associated with the other studied CVD outcomes in the complementary analyses except for IS in the FinnGen study in the MR-Egger method (OR,1.26; 95% CI, 1.07, 1.49; P = 0.006; **Figure 3**). No heterogeneity was observed for all reported results (**Table 2**) except for analysis for IS in the FinnGen consortium (P_Cochran’s Q_ = 0.042, **Table 2**). Furthermore, the P-values for intercept term in MR-Egger regression indicated that there was some evidence of horizontal pleiotropic effect in the analyses for IS and SVS in the FinnGen dataset (P_intercept_ = 0.006 for IS, P_intercept_ = 0.029 for SVS, **Table 2**), although the MR-PRESSO global test did not show any pleiotropy. For other CVD outcomes, both the P_intercept_ and P_MR-PRESSO global test_ suggested no significant overall horizontal pleiotropy (**Table 2**). In such cases, estimates from MR-Egger for IS and SVS in the FinnGen dataset were preferred. We further investigated the heterogeneity and pleiotropic effect of SNP rs532436, after including this SNP, the P-values for Cochrane’s Q and MR-PRESSO global test were dramatically decreased for all CVD outcomes except for SVS (**Supplementary Table 4**). Besides, the SNP rs532436 was identified as an outlier by the MR-PRESSO outlier test. These results verified that SNP rs532436 played a pleiotropic effect role and drove the heterogeneity in MR analyses with all six α-Klotho-associated SNPs.

**Table 2:** Sensitivity analyses of the causal associations of circulating α-Klotho levels with cardiovascular diseases.

Outcomes	Data sources	P_Cochran’s Q_	P_intercept_	P_MR-PRESSO global test_
Coronary artery disease	CARDIoGRAMplusC4D	0.477	0.537	0.474
	FinnGen	0.077	0.630	0.116
Atrial fibrillation	Nielsen et al.	0.162	0.321	0.103
	FinnGen	0.235	0.749	0.133
Heart failure	HERMES	0.667	0.443	0.586
	FinnGen	0.820	0.362	0.781
Any stroke	MEGASTROKE	0.778	0.274	0.771
	FinnGen	0.385	0.063	0.395
Ischemic stroke	MEGASTROKE	0.946	0.471	0.939
	FinnGen	0.042	0.006	0.052
Large artery stroke	MEGASTROKE	0.556	0.726	0.456
Small vessel stroke	MEGASTROKE	0.194	0.029	0.244
Cardioembolic stroke	MEGASTROKE	0.761	0.628	0.746

CARDIoGRAMplusC4D, Coronary ARtery DIsease Genome-wide Replication and Meta-analysis (CARDIoGRAM) plus The Coronary Artery Disease (C4D) Genetics; HERMES, Heart Failure Molecular Epidemiology for Therapeutic Targets.

## Discussion

In this two-sample MR study, we observed that genetically determined higher circulating α-Klotho concentrations were associated with decreased risk of AF. Whereas, no significant causal association of α-Klotho with CAD, HF, any stroke, IS, LAS, SVS, or CES was found. The results were consistent and robust across one or two of the different data sources and several complementary sensitivity analyses.

Our MR study corroborates results from previous observational studies which proclaimed a protective role of circulating α-Klotho on the risk of CAD and AF. Navarro-González et al. showed that reduced circulating Klotho concentrations were correlated with the presence and severity of CAD, independent of established cardiovascular risk factors (7). It was reported that circulating Klotho levels were inversely correlated with coronary artery calcification in patients with CAD (22). Likewise, Keles et al. explored the relationship between circulating Klotho levels and early atherosclerotic predictors (23). The results indicated that serum Klotho levels were inversely associated with the thickness of epicardial fat and carotid artery intima-media, and positively associated with the flow-mediated dilation of the brachial artery. Thus, they proposed that lower serum Klotho levels was a novel identified predictor of atherosclerosis. In addition, low Klotho and high FGF23 levels were associated with AF, indicating that Klotho may be protective against AF (24). Similarly, Mizia-Stec et al. reported that lower serum Klotho levels were correlated with episodes of AF, with serum Klotho levels comparable between patients with and without AF recurrence (907.9 ± 688.4 vs 686.4 ± 223.0 pg/ml in the left atrium, and 926.8 ± 616.9 vs 701.6 ± 245.3 pg/ml in the peripheral vein, respectively) (8).

Previous experimental studies showed that the potential beneficial role of circulating α-Klotho on CAD might be associated with protecting against endothelial dysfunction, arteriosclerosis, and calcification (25). Disruption of endothelial integrity was observed in Klotho-deficient mice, and Klotho-deficient cells showed enhanced Ca^2+^ influx and hyperactivity of Ca^2+^-dependent proteases, which could lead to vascular hyperpermeability and extensive vascular calcification (26). Klotho could modulate the effects of FGF23 on nitric oxide (NO) bioavailability, thus regulating endothelial function. As reported, FGF23 increased the expression of both Klotho shedding protease and soluble Klotho (27). With the presence of Klotho, FGF23 activates FGF receptor 1 and stimulates NO excretion *via* Akt-dependent activation of endothelial NO synthase in human coronary artery endothelial cells (27). It was also reported that preincubated with Klotho protein inhibited tumor necrosis factor-α-induced monocyte adhesion to human umbilical vein endothelial cells (HUVECs) and suppressed intracellular adhesion molecule-1 and vascular cell adhesion molecule-1 expression, suggesting that Klotho might play a role in modulating endothelial inflammation (28). In addition, pre-treatment with recombinant Klotho (200 pM) significantly prevented oxidized low-density lipoprotein-induced oxidative stress in HUVECs (29), indicating that Klotho might protect against CAD through its antioxidant effects. In the present MR study, although some evidence of suggestive protective effects of circulating α-Klotho on CAD was found in the CARDIoGRAMplusC4D and the meta-analysis but not in the FinnGen study, after accounting for multiple testing, Klotho was not associated with CAD. Thus, it is worthwhile to conduct further studies focusing on the association of α-Klotho with CAD and provide more mechanistic insights.

Circulating α-Klotho might exert a protective effect on AF through a variety of mechanisms. It was reported that Klotho-deficient mice showed phenotypes reminiscent of human aging; however, these phenotypes can be prevented and rescued by Klotho gene expression or protein supplementation (30). Considering AF is mainly attributed to senescence processes, Klotho appears to protect against AF through its anti-aging properties. Another plausible hypothesis is that Klotho might play an essential role in the normal function of sinoatrial function since Klotho was found expressed in the sinoatrial node and electrophysiological studies showed significant disrupted sinus node function in Klotho knock-out mice (31). Pulmonary vein (PV) triggers were recognized as an important source of ectopic beats for initiating AF from abnormal calcium handling. Hung et al. reported that Klotho (1.0 and 3.0 ng/mL) dose-dependently reduced beating rates and diastolic tensions of PV (32). L-type calcium current (I_Ca-L_), involved in the generation of action potential upstroke in pacemaker cells and responsible for calcium influx and calcium-induced calcium release, was found significantly suppressed by Klotho in isolated PV cardiomyocytes (32, 33). In addition, Klotho decreased the late sodium current, which resulted in attenuated calcium overload through reducing sodium-current-induced calcium influx (32). These results explained the mechanism of circulating α-Klotho’s protective role on AF to some extent; however, further clinical and experimental investigations are still warranted to figure out the direct effect of circulating α-Klotho on AF and whether α-Klotho could be a target for prevention of AF.

The correlation between α-Klotho and HF remains unknown so far. Taneike et al. found that soluble α-Klotho markedly increased in HF patients and decreased with successful treatment (34). However, another longitudinal study concluded that serum soluble Klotho was not correlated with the severity or progression of HF (35). Our MR analyses suggested that circulating α-Klotho levels were not causally associated with the risk of HF, adding robust evidence to the null association of Klotho with HF. When it comes to the association of circulating α-Klotho with stroke and IS, previous observational studies are limited to date and showed conflicting results as well. Lee et al. reported that elevated plasma Klotho levels were independently associated with good functional outcomes in patients with acute IS (36). Whereas, a recent prospective study found no neuroprotective effect of serum soluble α-Klotho, with α-Klotho not correlated with the severity of neurological deficits and long-term outcomes in IS patients (37). In our MR study, the estimates for SVS remained stable even we chose MR-Egger as the best approach (**Figure 3**). Whereas, estimate from MR-Egger for IS in the FinnGen consortium showed dramatical heterogeneity (P = 0.007) with the estimate from random-effects IVW method in the MEGASTROKE (**Figure 3**). Given that there was a larger number of cases in the MEGASTROKE (number of cases = 34,217) than in the FinnGen consortium (number of cases = 10,551) and no potential pleiotropic in the MEGASTROKE, the MR estimate for IS in the MEGASTROKE was preferred. Therefore, our study found limited evidence of causal a relationship between circulating α-Klotho levels and stroke, IS, or any IS subtypes, indicating that α-Klotho could not be regarded as a beneficial target in preventing these diseases.

Several limitations in this study should be noted. First, population restricted to European ancestry limited the generalization of our results to the population of other ancestries. Future studies on other ancestries are needed to extend our findings. Second, the possibility of pleiotropic effects of genetic instruments is a concern in MR analysis. However, we excluded the potential pleiotropic SNP (rs532436) to avoid horizontal pleiotropy bias. Besides, we performed several sensitivity analyses to address this issue, and the results turned out broadly consistent and no evidence of horizontal pleiotropy was detected by intercept in MR-Egger regression and global test in MR-PRESSO. Third, this MR study investigated the lifetime effect of circulating α-Klotho on CVD, and thus, the results could not be directly extrapolated to estimate the effect of any potential clinical interventions targeting circulating α-Klotho.

## Conclusion

Genetically predicted elevated circulating α-Klotho levels were causally associated with decreased risk of AF, indicating that it is important to monitor circulating α-Klotho levels during the aging process. Further clinical and experimental studies are warranted to figure out the underlying mechanisms and whether circulating α-Klotho could be treated as a target for preventing AF. No significant causal association between genetically predicted α-Klotho levels and risk of CAD, HF, stroke, IS, or IS subtypes was found.

## Data Availability Statement

The original contributions presented in the study are included in the article/**Supplementary Material**. Further inquiries can be directed to the corresponding author.

## Author Contributions

Study conception and design: XS and LZ. Data analyses: XS and LC. Draft preparation: XS, LC, and YH. Supervision of the study: LZ. All authors contributed to the article and approved the submitted version.

## Funding

This study was supported by the National Natural Science Foundation of China (no. 81873484) and the National Natural Science Foundation of China Youth Project (no. 82000316).

## Conflict of Interest

The authors declare that the research was conducted in the absence of any commercial or financial relationships that could be construed as a potential conflict of interest.

## Publisher’s Note

All claims expressed in this article are solely those of the authors and do not necessarily represent those of their affiliated organizations, or those of the publisher, the editors and the reviewers. Any product that may be evaluated in this article, or claim that may be made by its manufacturer, is not guaranteed or endorsed by the publisher.
